# Inpatient Geriatric Rehabilitation: Definitions and Appropriate Admission Criteria, as Established by Maltese National Experts

**DOI:** 10.3390/jcm11237230

**Published:** 2022-12-05

**Authors:** Francesca Muscat, Liberato Camilleri, Conrad Attard, Stephen Lungaro Mifsud

**Affiliations:** 1 Department of Physiotherapy, Faculty of Health Sciences, University of Malta, MSD 2090 Msida, Malta; 2 Statistics and Operations Research, Faculty of Science, University of Malta, MSD 2080 Msida, Malta; 3 Computer Information Systems, Faculty of ICT, University of Malta, MSD 2080 Msida, Malta

**Keywords:** geriatric rehabilitation, Delphi method, admission criteria, rehabilitation potential, inpatient rehabilitation

## Abstract

(1) Background: The importance of having an appropriate admissions system for geriatric rehabilitation is on the increase. However, the process of admitting patients to inpatient rehabilitation is a complex process. This is yet to be standardised across the European Union, as the approach to geriatric rehabilitation tends to vary from one Member State to another. (2) Objective: To discuss evidence-based practice with clinical experts, in order to define geriatric rehabilitation and admission criteria based on the Maltese population. (3) Method: The study entailed conducting four panel sessions using a purposive sample of thirteen local clinicians with extensive knowledge in clinical rehabilitation and healthcare management. A total of 48 items, based on the literature and clinical experience, were presented to the panel. Data analysis was done quantitatively and qualitatively, using IBM SPSS Statistics Version 24 and thematic analysis. (4) Results: The panel formulated a definition of rehabilitation, which shared common elements with the definition provided by the World Health Organization (WHO) and other sources/literature. The panel agreed on a list of eight criteria for appropriate inpatient geriatric rehabilitation admission in Malta. Consensus was also reached on: the need for a consultant-led multidisciplinary approach to assessment; the adoption of a standardised assessment processes for an equitable chance for all older adults assessed; the benefit of digital health in assessments; and the consideration that most patients would have some form of rehabilitation potential, depending on availability of resources. (5) Conclusion: Inpatient geriatric rehabilitation hospitals should have a unified strategy for rehabilitation services. The conclusions reached by the panel, could be useful in supporting the clinical evidence and establishing future rehabilitation guidelines and standards for inpatient rehabilitation.

## 1. Introduction

An ageing population is inevitably linked to increasing rates of morbidity and disability. By the end of 2020, 18.9% of Malta’s total population was aged 65 years and over [1]. The number of older adults has grown steadily over the past 10 years [1], necessitating increased management of healthcare spending and resource allocation.

Malta’s only state-run rehabilitation hospital admits, among other patients, older adults for inpatient rehabilitation. Since this hospital has a limited number of inpatient beds, appropriate admission criteria would be essential in ensuring that truly eligible patients are admitted.

Rehabilitation consultants and geriatric consultants, who assess patients for admission to inpatient rehabilitation, make daily clinical decisions on patients’ rehabilitation potential and eligibility for inpatient admission. There is currently neither an established systematic approach to assessing patients for rehabilitation potential nor a clinical guide for rehabilitation admission criteria [2,3,4]. Research indicates that various EU countries approach geriatric rehabilitation (GR) in different ways [4,5]. Although similarities could be noted across the existing literature, the particular healthcare service, culture, and other factors would cause local applicability of admission criteria to vary from one country to another [6,7,8,9]. Hence, the existence of pre-set admission criteria that could aid clinicians in selecting patients for inpatient rehabilitation, and admitting them to the correct ward, would be essential in ensuring that the most adequate level of care be provided to the patients [3,6,10,11]. A shared understanding of the best ways to serve the needs of these patient groups might encourage more international GR units to form a shared platform for learning to encourage the development of the service, globally.

In view of the above, the aim of this study was to pitch published evidence-based practices to the panellists and for them to discuss it, in order to define GR and admission criteria with reference to the Maltese population. Through the use of the Delphi method, we sought to involve a panel of national experts towards reaching a consensus on fundamental principles in GR.

## 2. Materials and Methods

### 2.1. Study Design

The Delphi method is a planned procedure that uses rounds of questions to gather experts’ opinions, without requiring face-to-face meetings [12]. The responses would be combined, analysed, and presented back to the panel of experts for further comment [13,14]. This process would be repeated until consensus was reached between most of the panel members, with the responses shifting towards the same ideas, thus suggesting stable results [11]. This Delphi study was completed over a total of four rounds.

### 2.2. The Panel

A set of criteria was established by the study team to identify the ideal experts to form part of the Delphi panel, taking into account the criteria required when forming a panel [13,15]. In order to ensure credibility of results, sociodemographic and educational characteristics were taken into consideration when choosing the panel. The selected experts held extensive knowledge in clinical rehabilitation and healthcare management. This was necessary, since admittance to rehabilitation also has a significant influence on healthcare finances and administration. The panel was composed of a heterogenous sample of experts, representative of the national field and hailing from different professions that constitute a multidisciplinary team (MDT) working in rehabilitation. This ensured that a broad platform of opinions would be represented. The experts were required to consent to keeping the information confidential and instructed to take all the time necessary to complete each round. Anonymity was crucial in ensuring an equal opportunity for each panellist to express their views, unconditioned by the identities or reactions of other panellists. Each panellist response had the same weighting score and was given equal importance at data analysis stage [11,16].

The research team identified thirteen experts through purposive sampling. These were individually contacted by e-mail and, upon expressing their interest in participating in the study, were provided with an information sheet and a consent form, which provided a detailed explanation of the aims of the study and what their involvement entailed. All of the selected experts that were invited consented to participate in the study. The panel of experts consisted of MDT professionals working in inpatient rehabilitation, namely: (1) geriatricians and rehabilitation consultants; (2) allied health professionals, including physiotherapists and occupational therapists; (3) nurses; and (4) social workers, all in management positions in rehabilitation, including that of older adults. These experts all worked on a full-time basis within the same Maltese rehabilitation hospital and had been fulfilling the same role for a minimum of five years.

In Malta’s rehabilitation hospital, members from the multidisciplinary team hold weekly meetings to discuss individual patient cases, progress, and plans for rehabilitation. These meetings are geriatric-consultant-led, and include the nurse, physiotherapist, occupational therapist, and social worker. Therefore, realistically, the panellists chosen for the study consisted of these professionals. This made the findings more contextually relevant.

### 2.3. The Rounds

SurveyMonkey, an online-survey platform, was used for all the rounds of Delphi. The surveys planned were sent individually to each panel expert, through an e-mail link. The participants were given one week to submit their responses; any panellist who had not yet filled in the questionnaires received a reminder in the week following the set deadline. Maintenance of participation levels between 50 and 80% was necessary to ensure that a valid consensus could be reached [12,17]. In order to encourage participation, the number of rounds was set prior to the sessions, with the panel being informed from the outset [11,12]. All experts completed the questionnaires provided in all four rounds.

The aim of the Delphi survey was to define geriatric rehabilitation (GR), discuss admission criteria for inpatient GR, discuss the composition of the MDT, and discuss the use of digital technology to help guide clinicians to make consistent, transparent decisions on GR admission, for the Maltese population. The statements presented in the rounds were mostly based on evidence regarding rehabilitation and GR potential, and on responses given by the panellists from previous rounds.

The first round of Delphi, served to create a baseline for the panel, in order to ensure that the points for discussion and the theme of the study were clear and concise [13,14]. Participants were asked both close-ended and open-ended questions, to also allow them a degree of freedom with their answers. The close-ended questions, varied from rating agreement using a Likert scale, numerical rating, and tick-as-many-as-apply—all of which related to rehabilitation potential (RP) in older adults and its impact on admission to inpatient GR. As the rounds progressed, the questions were presented on a Likert scale, ranging from ‘strongly agree’ to ‘strongly disagree’.

**Figure 1:**
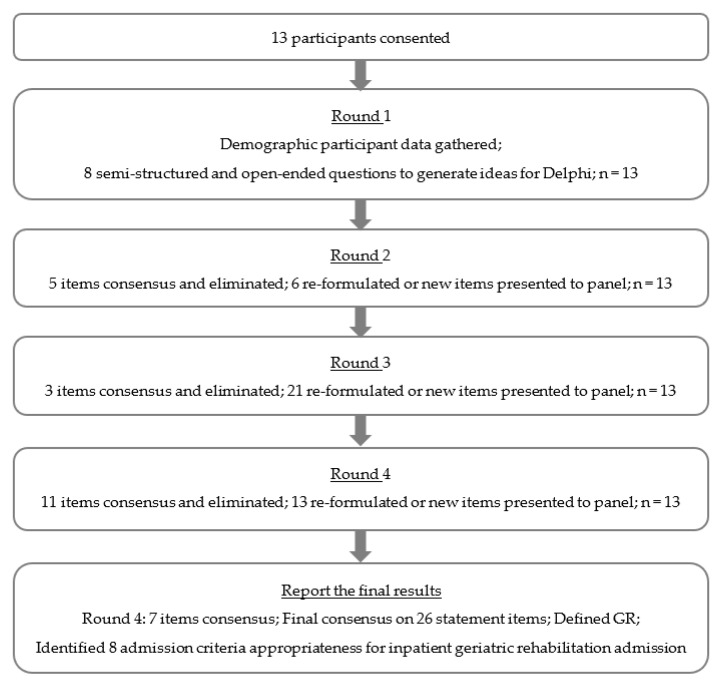
Flowchart showing Delphi rounds.

The first round concerned: rehabilitation definitions; domains that affect RP; admission criteria; assessment measures; and outcomes of rehabilitation—all of which were initially compiled from the literature. This was an introduction that served as the foundation of the questions in the remaining rounds. Indeed, the second questionnaire was created using the experts’ responses from the first Delphi round, eliminating the items that had already attained a high level of homogeneity, and maintaining the ones that had not shown a sufficient level of consensus. The ideas generated by the experts in their remarks from the first round were taken into consideration when these elements were revised and reintroduced to the panel in the second round. New multiple-choice questions were also included. A total of 48 items were presented to the panel during the rounds. Figure 1 below, provides further detail on the items presented. Following each close-ended question, an open space was provided, through which the panellists could give reasons for their answers, thus allowing their responses to be more effective and complete [11,12,13].

Consensus was considered to have been achieved if at least 70% of the panellists responded to the same statement from among a choice of ‘strongly agree’ and ‘agree’, or ‘strongly disagree’ and ‘disagree’ [3,11]. This meant that if the expert consensus gravitated towards agreement with the given statement, the item was considered as an appropriate admission criterion. On the other hand, if the experts reached consensus on disagreement with a statement, the item was considered as an inappropriate admission criterion [3,12]. This percentage of consensus was calculated using mean values and confidence intervals, and 70% was considered as the final conclusion of the Delphi method. Quantitative data analysis was performed using the computer package IBM SPSS Statistics version 24 (SPSS, Inc., Chicago, IL, USA).

On the other hand, qualitative data analysis was carried out manually. Each question was evaluated thoroughly, and the main points and common themes were grouped together. This process is called thematic analysis, as described by Bryman [18].

### 2.4. Ethical Considerations

This study was reviewed by, and received approval from, the University of Malta Research Ethics Committee. Each panellist was given one code which they were required to apply when completing the rounds. A second code was allocated to each participant, which was only known by the researcher [12]. This code was a pseudonym given to each expert, to be used when referring to them during data reporting. These codes were password-protected, to protect the identity of the panellists. Informed written consent was required prior to the commencement of the rounds. In the information sheet, panellists were informed that they had the right to withdraw from participation at any point during the rounds, without any implication or consequence. The information sheet also stated the GDPR and national legislation regarding data protection.

## 3. Results

The results of the rounds are presented in sections. The first section (Section 3.1) provides the panellists’ information. The following four sections (Section 3.2, Section 3.3, Section 3.4, Section 3.5) will manifest the development of the discussions within the Delphi process. Detailed report analysis is shown in Section 3.6 (open-ended statements) and Section 3.7 (close-ended statements).

### 3.1. Participant Demographics

The panel consisted of thirteen experts—53.8% male and 46.2% female—with 38.5% aged between 45 and 54 years; 46.2% held a master’s degree, while 46.2% worked in rehabilitation for over 20 years, across clinical and managerial roles. All experts were Maltese nationals. Further demographic and educational characteristics of the participants is presented in Appendix A.

### 3.2. Delphi Round One

The first round involved the circulation of two questionnaires, one aimed at collecting the experts’ demographics and one covering questions regarding the topic under discussion. The latter was composed of eight questions, one of which was quantitative in nature. Most questions showed similarities and convergence, and the aim of this round was to generate ideas and link the responses to the literature [2]. Most responses agreed with existing literature, with the main themes being formulated in statements and reintroduced in the second round for consensus within the panel.

### 3.3. Delphi Round Two

The second round consisted of six questions, one of which was open-ended. The first three questions were reproduced from the first round. The remaining three questions were first introduced in this round. The participants were required to respond to more defined statements regarding definitions of rehabilitation, which domains have the highest implication on rehabilitation, and which outcome measures should be used to assess these domains. The round also considered which clinician/s from the multidisciplinary team (MDT) would be the most suitable for conducting the assessment, and if digital technology could aid this assessment process in any way.

Percentage consensus was calculated, with three questions showing convergence, and hence were eliminated from subsequent rounds. Three questions were reintroduced in the following round, even though there were similarities in the responses. These similarities emerged through the ‘additional comment’ field following the questions, as some panellists clarified the statements that merited further discussion and thus offering higher convergence.

### 3.4. Delphi Round Three

The questionnaire in the third round consisted of ten questions, which included sub-questions to a total of twenty-one items. This round sought to gather the panel’s views on the MDT involvement in assessment, the admission criteria, and the use of digital technology in the assessment process. The questions that were reformulated from the second round were intended to narrow down the responses, aiming for a more concise and homogenous answer from the panel.

A good degree of convergence was achieved in three of the five questions asked on the MDT approach to assessment, in three of the four questions asked on digital technology use in assessment, and in five of the twelve questions asked on admission criteria to inpatient rehabilitation. This meant that the following round required further clarification and reformulation of statements regarding admission criteria.

### 3.5. Delphi Round Four

The questionnaire in the fourth round consisted of four questions, with sub-questions to a total of thirteen items discussed. These included re-submitted questions on the MDT assessment process and on digital technology involvement, and also re-introduced and new questions on admission criteria to inpatient rehabilitation. The aim of this round was to continue evaluating the panel’s views on which patients should be considered eligible for inpatient rehabilitation, and which would benefit from other services instead.

Convergence was noted in most replies. However, four items did not deliver a homogenous reply and the responses were distributed throughout the five-point Likert scale, and thus were not conducive to reaching a final conclusion. This was noted in statements that did not reach consensus in the previous round, and not in new statements introduced. For this reason, and since this was the final round of the Delphi survey, these items were excluded from the list of appropriate admission criteria. A summary of the survey results was sent to the panel. 

### 3.6. Summary of Open-Text Questions

A total of 20.8% of the questions asked were open-ended in nature. The main themes were extracted, and the statements were formulated and presented to the panel for discussion. Any themes that obtained immediate consensus were eliminated from subsequent rounds.

In the first round, the participants were presented with standard definitions of rehabilitation, geriatric rehabilitation (GR), and rehabilitation potential (RP), and were asked to comment on the statements. A total of 84.6% agreed with these definitions but added to the description of rehabilitation potential. The remaining 15.4% maintained that there should be no distinction between rehabilitation and GR. The results were collated together and one statement to define rehabilitation was sought and presented to the panel in the second round, which subsequently obtained a 92.3% consensus.

The panellists were asked about domains that affect RP and the open-ended follow-up question was to ‘list any other factors that affect rehabilitation and a patient’s potential’. A total of 76.9% maintained that the patient’s character, motivation, and willingness to undergo rehabilitation would play a key role in the patient’s performance, and thus the outcome. The comments also included the mental state of the patient, including depression and anxiety, and the ability to participate in, and benefit from, rehabilitation.

Three questions achieved consensus in the first round, which aided in the formulation of the admission criteria statements that were presented in Rounds 3 and 4. The responses obtained in the close-ended questions were compared to these results, and are presented in the following section.

In contrast to the above, two questions did not reach consensus during the first presentation of the open-ended question, and required clarification and re-discussion in the ensuing round. These questions concerned which outcome measures should be used when assessing patients for GR inpatient admission. There were a number of outcome measures mentioned in this section, none of which were mentioned by the majority. In total, 34 outcome measures were mentioned, with the most commonly mentioned being the Barthel index or BI (14.7%), the functional independence measure or FIM (14.7%), the mini mental state examination or MMSE (11.8%), the clinical frailty scale or CFS (11.8%), and the Montreal cognitive assessment or MoCA (8.8%). The responses obtained were compared to the existing literature [2] and a list of outcome measures were presented to the panel in the second round. The said list was based on the highest-rated outcome measures from the highest-rated domains mentioned by the panel. The outcome measures presented to the panel were the BI, FIM, MMSE, Charlson comorbidity index (CCI), and the timed up and go test (TUG). Consensus was reached in this statement with 92.3% agreement.

The remaining two questions were free-text questions, which allowed the panellists to comment freely on anything that was discussed during the panel sessions. These were asked during Rounds 3 and 4. The panellists commented on various topics of the survey, including the benefits of a standardised approach to patient assessment, the benefits and risks of using digital technology to compile and store assessment data, and the challenges faced by clinicians when trying to identify the most adequate admission criteria. In these two questions, none of the themes extracted showed convergence. Hence, these responses were used as a basis for further statements or to enhance the robustness of already discussed statements.

Notwithstanding the above, similarity was noted within the panel in mentioning that GR assessment should be patient specific, and depending on individual characteristics, patient motivation, and rehabilitation potential. In view of clinical complexity and the frailty of referred patients, the panel questioned the ability of clinicians to predict rehabilitation outcomes in an effective manner, in the absence of a standardised approach to assessment, or the presence of only one clinician, assessing the patient and making the decision alone.

### 3.7. Summary of Statement Results

A total of 79.2% of the statements were close-ended in nature. Convergence was noted in 52.6% of the statements, more specifically: 2.6% on rehabilitation definitions and potential; 7.9% on outcome measures and domains; 10.5% on composition of the MDT; 18.4% on statements on admission criteria; and 13.2% on the use of digital technology in the assessment process.

The panellists were presented with a list of domains, extracted from the existing literature [2], and were asked to rate each domain according to its impact on RP. Following two rounds, the panel agreed (92.3%) that the main domains affecting RP were: cognition, activity of daily living function, comorbidities, mobility, social support, and behaviour.

The MDT involvement in patient assessment constituted 15.8% of the statements, of which 10.5% reached convergence. The panel agreed that the admitting team should consist of a consultant geriatrician or rehabilitation specialist, a physiotherapist, an occupational therapist, and a social worker, with the potential addition of a speech and language pathologist, psychologist, dietician, and other pertinent professionals, depending on the individual patient’s needs. The panel responses are shown in Table 1. The MDT is consultant-led, but no single professional in the MDT had the necessary skills to carry out the assessment alone, thus suggesting the necessity of the involvement of more than one clinician during GR assessment.

The remaining 5.3% of the MDT statements showed a lack of agreement on the involvement of nurses. The panel was asked whether nurses should be involved in GR patient assessment, with 46.2% of the panel being in agreement. Since consensus was not reached, the statement was represented to the panel in the following round, noting an increase to 61.6% agreement. The panel showed conflicting responses that the nurse should be involved mostly in cases where the patient would require specialist nursing, such as wound management or pain management, on a referral basis.

The panellists were asked what they considered to be a ‘good’ outcome of rehabilitation and a ‘poor’ rehabilitation potential. A total 46.2% of the panel maintained that a good outcome of rehabilitation would occur when the patient could be reintegrated within the community, in previous housing or living with a family member, while 30.8% considered a patient reaching their maximal potential as a good outcome. Another 30.8% mentioned reaching patient and caregiver’s goals and objectives of rehabilitation. In contrast, most panellists (84.6%) considered poor prognosis and multiple comorbidities to be the lead determinants of poor RP. The panel argued that decreased medical stability tended to lead to a decrease in ability to co-operate during therapy sessions, thus rendering rehabilitation more challenging. Co-operation would be further decreased in the presence of impaired cognitive function, as mentioned by most panellists (61.5%), and possibly leading to a lack of willingness and motivation to adhere to treatment sessions. A lack of social support was considered by 38.5% of the panellists, along with 15.4% believing that the level of independence prior to the event that led to hospitalisation would have an impact on the success of the rehabilitation phase.

Based on the above-mentioned responses, the panellists were then asked to discuss which admission criteria they deemed necessary for inpatient GR. Table 2, Table 3 and Table 4 demonstrate the criteria that achieved high or average convergence, and criteria that did not reach consensus. The four statements that obtained more than 50% convergence, but did not reach consensus (Table 3), had stronger consensus in the open-ended questions asked in previous rounds. The panellists commented on the importance of seeking patient level of support, both prior to and in the wake of the acute episode, and also seeking the relatives’ or main carer’s wishes and availability to continue providing the necessary support. The panel emphasised the importance of the involvement of the caregiver to aid the integration of the patient back into the community. The panellists also commented on the importance of assessing the patient and evaluating the level of therapy and rehabilitation required, depending on whether the patient would still be able to live functionally in the community or otherwise. Therefore, although these statements did not obtain at least 70% agreement [3], most panellists also mentioned these points in open-ended statements.

Two particular statements represented a higher disagreement level, yet did not reach dis-consensus. These statements addressed the medical stability of the patient and read as follows: ‘Has a need for daily nursing and medical care’ and ‘Multiple admissions in the previous year’. Therefore, by not reaching a suitable level of agreement, the panel shifted towards admitting patients who would be sufficiently medically stable to withstand intensive rehabilitation programmes.

Another statement that remained inconclusive concerned the cognitive status of the patient. There was no agreement with the statement that patients with moderate-to-severe cognitive impairment should be excluded from GR. While some panellists maintained that patients with cognitive impairment could still be rehabilitated if the staff would be well trained and the environment adequate in accommodating their needs, others maintained that if the cognitive impairment were to be a hindrance to rehabilitation compliance, or the impairment should cause a challenge for the relatives if the patient were to be rehabilitated, then the patient should be excluded from inpatient GR. Delirium and premorbid cognitive status were also mentioned as important considerations for GR admission. Furthermore, panellists mentioned the importance of assessing cognitive status to be in the best position to plan treatment sessions accordingly.

The panellists were also presented with questions regarding the use of digital technology in the process of patient assessment for admission to GR. Table 5 summarises the statements provided and the degree of convergence.

The panel agreed that a digital standardised assessment should not be the final and definite decision that would replace clinical judgement. Nevertheless, it should serve as an assistive tool that would ensure that the patient be assessed in a holistic manner, identifying patient needs and expectations, and supporting the better planning of treatment and rehabilitation care. A total of 15.4% of the panel highlighted the point that a standardised digital approach would offer greater transparency in the assessment process, and would allow increased ease of communication with other healthcare entities and professionals, as well as the patients themselves and their caregivers.

## 4. Discussion

This study was the first in Malta to employ the Delphi method in seeking to identify what is understood by rehabilitation and which admission criteria are the most appropriate for inpatient geriatric rehabilitation (GR). There was a 100% response rate in all four rounds, with all the panellists replying to the questionnaires within the set time frame. Consensus was not reached on: the nurses’ involvement in inpatient admission assessments and on admission criteria concerning patient cognitive level; previous long-term care applications; the need for daily nursing care; and the number of admissions to hospital in the previous year.

Having stated the above, similarities were noted in the responses, even when the panel chose ‘neutral’ in their scale response. This suggests that there were implied shared criteria of acceptability among the panellists, despite there being no current standardisation of assessment. By the end of the panel sessions, the experts agreed on a list of eight criteria for appropriate inpatient GR admission (Table 6). Admission to inpatient GR could be considered should the patient meet the majority of these criteria.

The definition of rehabilitation that was agreed upon among the panellists was as follows: ‘Rehabilitation is a multidisciplinary approach of interventions, aimed to maximise functional independence and facilitate psychosocial adjustments to residual disability, to allow a patient to function successfully in the community’. This was similar to the definition offered by the WHO [19] and that was put forward in the other literature [9,20]. It was concluded by the panel that rehabilitation potential (RP) is a complex process of clinical assessment and judgement, which would affect the type of rehabilitation for the patients. Some panel members maintained that every patient would have some degree of potential, which is patient-specific, and conditioned by the patient’s functional abilities, aims, and expectations. Hence, a watertight definition of RP that would be clinically useful proved challenging to produce. Wade [20] and the Cochrane Rehabilitation initiative cited in Negrini et al. [21] have also investigated definitions of rehabilitation and found over 100 definitions, similarly inferring that this term was difficult to define. Similarly, these sources also concluded that the resulting definition would require re-evaluation within a relatively short period of time. Grund et al. [9] aimed at gathering EU consensus on principles for GR, one of which was defining this term. The study produced a similar definition, but also made reference to national policies, resource availability, and setting [9].

In the current study, it was ultimately agreed that GR assessment was an extensive and detailed process that would require a multidisciplinary approach. This would help structure a patient-oriented rehabilitation plan. Inpatient GR services should evaluate the assessment process on a regular basis, to detect any flaws, or areas of improvement. This could be based on evaluation of: duration of stays in hospital; rates of mortality during rehabilitation; fluctuating numbers of admissions and patients referred for long-term care institutions; and functional abilities in the community with follow-ups [11]. The assessing team and case managers should use outcome measures and standardised approaches to evaluate adequately the patient’s functioning and participation. The panel was in agreement that the GR assessment should be done by the MDT, and be consultant-led, in line with the GR team structure [9]. This meant involving the geriatric consultant or rehabilitation specialist, a physiotherapist, an occupational therapist, and a social worker. The panel did not reach consensus on the involvement of nurses in the assessment.

It appeared that some of the experts on the panel expressed their views not solely based on their clinical expertise and knowledge, but were also influenced by the administrative and managerial problems encountered in the day-to-day running of rehabilitation in Malta. The panel indicated that the assessment process should be more standardised and thorough, portraying some similarities with the comprehensive geriatric assessment (CGA) model [9,22,23]. However, the panel also noted that, while being the ideal, this process could at times be compromised by the number of referrals received and the need to make rapid and single-time point decisions, to favour admission. Thus, a patient’s admission to GR might be subject to the availability of resources at the time of the assessment. This consideration is consistent with the existing literature [11,23,24,25]. Moreover, since available resources could be limited, and thus denied to patients who could have benefitted from rehabilitation, setting a standardised GR assessment tends to remain challenging.

Although the majority of the panel maintained that RP and admission criteria should be based on improving the patient’s functional abilities, a few panellists also mentioned the importance of offering rehabilitation even in cases requiring higher levels of medical, adaptive, or palliative care [24,25]. Further research would be required to better understand the role of inpatient GR in palliative care patients or very frail patients. The panel also agreed that motivation would be a crucial component of a GR patient assessment. Motivation increases patient compliance to intensive rehabilitation programmes, thus increasing the chances of a successful outcome.

The panel did not reach consensus on admission criteria regarding the cognitive level of the patient. The panellists had conflicting responses as to whether patients with dementia or cognitive impairment tended to have lower RP and lower compliance rates to treatment, as was noted in at least one study [26]. In contrast, Goodwin and Allan [27] have stated that such a conclusion was based on clinicians’ assumptions, rather than proven research. In fact, the consensus study in Grund et al. [9] showed that presence of cognitive impairment should not exclude patients from GR, even if this required further planning and tailoring of the rehabilitation treatment plan. Therefore, further research focusing on RP in cognitively impaired patients would be beneficial in reaching consensus and appropriate admission criteria in this regard [5].

Finally, a number of panellists also mentioned that no patient should be denied rehabilitation, and that patients should be equally eligible for inpatient GR, irrespective of their premorbid or current condition. Nevertheless, it was also pinpointed that the available resources presented ethical challenges and the need for prioritisation of service allocation. This may be viewed as a contradiction to the comments provided above.

### Strengths and Limitations

It should be noted that the use of the Delphi method approach is not intended as an alternative to rigorous scientific research, but to inform it [12,13]. Consensus from a Delphi process does not imply that the proper definition or solution has been established. However, it indicates that a consensual response has been achieved among the national experts included in this study. This research seeks to provide an opinion on rehabilitation and its potential, and this may change over time [12]. Malta is a small island, having one state rehabilitation hospital. The clinicians were chosen based on well-known expertise and professional seniority. Indeed, this could be seen as a strength of the study as panellists can be seen as influential as change agents.

Sample size recommendations varied across literature, with minimum numbers including five, ten, or twelve panellists to be sufficient to enable consensus [13,28,29]. Thus, the choice of thirteen panellists was chosen for this study. The panellists chosen were highly experienced clinicians working in rehabilitation, across professions making up the MDT. The panel was set up according to the researchers’ knowledge of the potential contribution of the chosen experts to the study. Hence, this selection was not exhaustive in terms of pertinent clinicians working in the field. The heterogenous sample of clinicians on the panel contributed to the study’s strengths by widening the scope of the applicability of the findings, by providing broader insights on rehabilitation for the older adult population. The study took careful consideration of the panel’s clinical experience in rehabilitation towards ensuring high standards of knowledge, clinical reasoning, and arguments presented.

The anonymity and confidentiality employed during the rounds helped minimise the bias in the panel responses, which may have otherwise been influenced by existing relationships between the panel and the researchers involved. The Delphi method reduced the possibility of any panel members being reluctant to express their views if these contradicted others or if some panel members were more expressive than others. Existing literature has also highlighted this advantage of the Delphi method [12,14,17].

## 5. Conclusions

This study proposes that geriatric rehabilitation (GR) and its assessment is a complex decision-making process through which clinicians are required to make clinical judgements on the patient’s potential and expected outcomes if admitted to an inpatient GR programme.

The panel agreed about the need for a consultant-led multidisciplinary approach to rehabilitation assessment and management. The inclusion of other stakeholders, such as the patients themselves and their caregivers, could offer a wider view of rehabilitation in older adults. This research focused solely on clinicians working in the field of rehabilitation, but a further step could be taken to include these stakeholders in future research. The panel believed that, while all patients had some form of rehabilitation potential, the provision of rehabilitation would depend on the wishes of the patients and caregivers, and on the resources available.

The conclusions obtained by this panel could be useful in supporting the existing clinical evidence and establishing rehabilitation guidelines and standards for inpatient rehabilitation. Inpatient GR hospitals should have a unified strategy for rehabilitation services. Considering that the Delphi panel findings represent local expert opinions, rather than more far-reaching scientific evidence, further research to solidify the conclusions would be crucial [11]. Nevertheless, the compiled findings could be a useful starting point for the future support of inpatient GR services.

## Figures and Tables

**Table 1:** MDT involvement in GR assessment.

Clinician	Percent Agreement (%)	Number of Rounds for Consensus (n)
Doctor/consultant geriatrician/rehabilitation consultant	100	1
Physiotherapist	84.6	1
Occupational therapist	84.6	1
Social worker	77.0	2
Nurse	61.6	2

**Table 2:** Appropriate criteria for inpatient GR Admission. (Pt = patient; PADLs = personal activities of daily living; IADLs = instrumental activities of daily living).

Criteria	Convergence	Percent Agreement (%)	Number of Rounds for Consensus (n)
Pt is in a phase of medical stability, and able to withstand the rehabilitation plan	High	100	1
Pt requires an intensive rehabilitation programme, following hospitalisation for an acute event	High	100	1
Pt needs an integrated MDT approach (the input of a minimum of two allied health professionals)	High	92.3	1
Pt has a favourable rehabilitation prognosis	High	92.3	1
Pt experienced a change from pre-admission level of function, to current level of function, in PADLs and IADLs	High	84.6	1
Pt is motivated and co-operates in rehabilitation plan	High	84.6	1
Pt shows functional improvement from acute admission to rehabilitation referral assessment time	Average	76.9	2

**Table 3:** Criteria for inpatient GR admission that had more than 50% agreement, but not consensus. (Pt = patient).

Criteria	Convergence	Percent Agreement (%)	Number of Rounds (n)
Pt previously had adequate physical mobility	Average	69.3	2
Pt otherwise unable to manage at home	Average	61.6	2
Pt is in need of social-care review, for home environment or social support	Average	61.6	2
Pt is supported by relatives, who are able and willing to help	Average	53.9	2

**Table 4:** Criteria for inpatient GR admission that was inconclusive. (Pt = patient).

Criteria	Percent Agreement (%)	Percent Neutral (%)	Percent Disagreement (%)
Pt did not have multiple admissions in the previous year (acute or rehabilitation hospitals)	30.8	23.1	46.2
Pt has a need for daily nursing and medical care	15.4	38.5	46.2
Pt is not scoring moderate-to-severe cognitive impairment	38.5	30.8	30.8
Pt does not have a community application for long-term care	30.8	38.5	30.8

**Table 5:** Digital technology statements presented to the panel.

Digital Technology Statements	Convergence	Percent Agreement (%)	Number of Rounds for Consensus (n)
A digital patient assessment, providing real-time prediction of rehabilitation potential, is a guide to clinicians to make consistent, transparent, patient-centred, and evidence-based decisions about inpatient rehabilitation admission.	High	100	2
I would feel comfortable using a tablet.	High	100	1
A digital patient assessment should be linked to other healthcare networks to easily provide patient demographic data.	High	100	1
A digital format of the assessment is more convenient and easier to use, than in a physical format.	High	84.6	2

**Table 6:** Appropriate admission criteria.

	Criteria
1	Patient is medically and cognitively stable. This stability is sufficient to avoid undermining the rehabilitation plan, thus safety is guaranteed. Could withstand an intensive rehabilitation programme.
2	Functional improvement is conceivable. Patient shows favourable rehabilitation prognosis and improvement from acute to rehabilitation referral time.
3	Requires an integrated IDT/MDT approach, and needing the input of more than two rehabilitation professionals.
4	Experienced a decrease in self-sufficiency. Acute episode resulted in a decline in IADLs or PADLs. Patient is no longer able to mobilise or take care of self; unable to manage at home.
5	Patient previously enjoyed adequate physical mobility.
6	Manifests signs of motivation and co-operation to rehabilitation admission and programme.
7	Patient is supported by relatives. Relatives and patient’s plans are addressed.
8	If stable but functional improvement is not conceivable: rehabilitation intervention can be focused on the reduction of level of disability. Patient receives education on self-management.

## Data Availability

Not applicable.

## Appendix A. Panel Characteristics

**Table A1:** Participant demographics and characteristics.

Characteristics		Percent (%)
Gender	Male	53.8
	Female	46.2
Age	25 to 34	23.1
	35 to 44	15.4
	45 to 54	38.5
	55 to 64	23.1
Job title	Doctor	38.5
	Nurse	15.4
	Physiotherapist	15.4
	Occupational therapist	15.4
	Social worker	15.4
Level of education	Bachelor’s degree	7.7
	Post-graduate diploma	7.7
	Medical doctor	15.4
	Master’s degree	46.2
	Doctorate/PhD	15.4
	Other	7.7
Years of experience in rehabilitation	0 to 4	0.0
	5 to 9	38.5
	10 to 14	7.7
	15 to 19	7.7
	20 or over	46.2
